# Renal Cell Tumors: Understanding Their Molecular Pathological Epidemiology and the 2016 WHO Classification

**DOI:** 10.3390/ijms18102195

**Published:** 2017-10-20

**Authors:** Kentaro Inamura

**Affiliations:** Division of Pathology, The Cancer Institute, Japanese Foundation for Cancer Research, 3-8-31 Ariake, Koto-ku, Tokyo 135-8550, Japan; kentaro.inamura@jfcr.or.jp; Tel.: +81-3-3570-0111 (ext. 5604); Fax: +81-3-3570-0558

**Keywords:** histology, immunohistochemistry, kidney, genetic alteration, molecular pathology, renal cell carcinoma

## Abstract

Accumulating evidence suggests that renal cell tumors represent a group of histologically and molecularly heterogeneous diseases, even within the same histological subtype. In accordance with the increased understanding of the morphological, immunohistochemical, molecular, and epidemiological characteristics of renal cell tumors, the World Health Organization (WHO) classification of renal cell tumors has been modified. This review provides perspectives on both new and current subtypes of renal cell tumors, as well as on the emerging/provisional renal cell carcinomas in the new 2016 WHO classification, which focuses on features of their molecular pathological epidemiology. The WHO classification will require additional revisions to enable the classification of renal cell tumors as clinically meaningful subtypes and provide a better understanding of the unique characteristics of renal cell tumors.

## 1. Introduction

Renal cell tumors represent a group of histopathologically and molecularly heterogeneous tumors, with different sets of genetic and epigenetic abnormalities [1,2,3,4,5,6,7,8,9,10,11,12,13,14,15,16,17,18,19,20,21,22,23,24,25,26,27]. An increased understanding of the morphology, immunohistochemistry, genomics, and epidemiology of renal cell tumors has resulted in the identification of novel features in their molecular pathological epidemiology. On the basis of these features, the classification of renal cell tumors has recently been revised and published in the 2016 World Health Organization (WHO) classification [1]. The revisions reflect the increased understanding of the characteristic features of renal cell tumors. This review introduces and briefly summarizes the molecular pathological epidemiologic features of both new and current subtypes of renal cell tumors, as well as of emerging/provisional renal cell carcinomas (RCCs), in the 2016 WHO classification.

## 2. The 2016 WHO Classification

The revised WHO classification is based on advances in the understanding of newly identified characteristics of the molecular pathological epidemiology of renal cell tumors. The majority of the International Society of Urological Pathology (ISUP) Vancouver classification of renal neoplasia [28] was adopted for the revised 2016 WHO classification of renal cell tumors [1]. Perspectives on both new and current subtypes of renal cell tumors, as well as on emerging/provisional RCCs in the new 2016 WHO classification, are described below.

**Figure 1:**
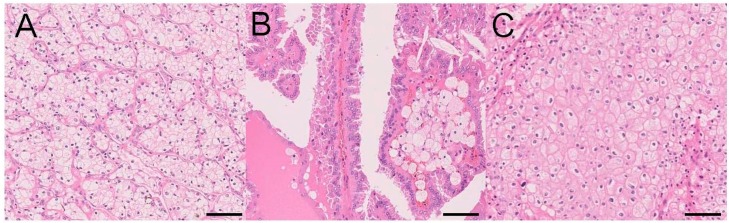
Morphology of the major subtypes of renal cell carcinoma (RCC) (hematoxylin and eosin staining; scale bar, 100 µm). (**A**) Clear cell RCC; (**B**) papillary RCC; and (**C**) chromophobe RCC. Clear cell RCC typically consists of clear cells with thin-walled, staghorn-shaped vasculature (**A**). Papillary RCC is typically composed of papillae formed by fibrovascular cores that often contain foamy macrophages (**B**). Chromophobe RCC typically contains a mixture of eosinophilic and clear cells with voluminous cytoplasm, perinuclear clearance, and well-defined cell borders, displaying a solid, sheet-like pattern (**C**).

The various subtypes of renal cell tumors are based on descriptive or characteristic features (Table 1) [1]. The major subtypes are clear cell RCC (CCRCC) (Figure 1A), papillary RCC (PRCC) (Figure 1B), and chromophobe RCC (ChRCC) (Figure 1C), which comprise 65–70%, 15–20%, and 5–7% of all RCCs, respectively. The names of these subtypes are based on their predominant cytoplasmic (cellular) and staining features (e.g., CCRCC, ChRCC, and renal oncocytoma), structural and morphological features (e.g., PRCC), and combinations of these features (e.g., clear cell papillary RCC (CCPRCC)). Other terms for renal cell tumors are based on the anatomical location of the tumor (e.g., collecting duct and renal medullary carcinomas), association with renal disease (e.g., acquired cystic disease-associated RCC (ACD-associated RCC)), pathognomonic molecular alterations (e.g., microphthalmia transcription factor (MiT) family translocation RCC (TRCC) and succinate dehydrogenase-deficient RCC (SDH-deficient RCC)), and familial predisposition (e.g., hereditary leiomyomatosis and RCC-associated RCC (HLRCC-associated RCC)) [1].

## 3. Current Major Subtypes

In this section, the current major subtypes of renal cell tumors in the 2016 WHO classification are briefly summarized, with a focus on their molecular pathological epidemiology. Their characteristic features are summarized in Table 2. 

### 3.1. Clear Cell RCC (CCRCC)

CCRCCs comprise 65–70% of all adult RCCs. Macroscopically, CCRCCs appear typically yellow, with evidence of necrosis and hemorrhage. Microscopically, CCRCCs consist of clear/eosinophilic cells with thin-walled, staghorn-shaped vasculature. The cytoplasm of CCRCCs is typically clear from an abundant accumulation of glycogen and lipids. Immunohistochemically, CCRCCs are typically positive for CAIX and CD10, and negative for CK7 and AMACR. Genetically, a loss of function of *VHL* at 3p25-26 is nearly ubiquitous. One copy of *VHL* is either mutated or silenced in 90% of sporadic CCRCCs, whereas another copy is typically lost through 3p deletions, according to the comprehensive molecular profiling of CCRCCs by The Cancer Genome Atlas (TCGA) [2]. The biallelic loss of *VHL* allows for the inappropriate stabilization of hypoxia-inducible factors (HIFs), which results in a proangiogenic gene expression signature that is critical to the carcinogenesis of CCRCC [29,30]. According to the TCGA, CCRCCs are characterized by recurrent mutations in the PI3K/AKT/MTOR pathway (a potential therapeutic target), mutations in *SETD2* (associated with widespread DNA hypomethylation), and mutations involving the SWI/SNF chromatin remodeling complex (*PBRM1*, *ARID1A*, and *SMARCA4*). Aggressive CCRCCs demonstrate a metabolic shift [2].

### 3.2. Papillary RCC (PRCC)

PRCCs comprise 15–20% of all adult RCCs. PRCCs have been associated with a better prognosis than CCRCCs. Furthermore, type 1 PRCCs are associated with a better prognosis than type 2 PRCCs. However, recent evidence suggests that metastatic PRCC patients fare worse than metastatic CCRCC patients [31]. Hereditary PRCC syndrome with germline mutations in *MET* is associated with type 1 PRCCs. Macroscopically, these tumors usually display a friable appearance with evidence of necrosis and hemorrhage. Microscopically, PRCCs have been traditionally subclassified into two subtypes. Type 1 PRCCs have papillae, covered by cells with scanty cytoplasm and nuclei, arranged in a single layer on the papillary cores, with or without foamy macrophages. Type 2 PRCCs are typically composed of cells with abundant eosinophilic cytoplasm and large pseudostratified nuclei that line papillary structures with true fibrovascular cores, with or without foamy macrophages. A subset of PRCCs has a mixed histology. Immunohistochemically, PRCCs are typically positive for CD10, CK7, and AMACR, and negative for CAIX. Of note, CK7 positivity is more prominent in type 1 PRCCs and is often decreased in type 2 PRCCs. Therefore, CK7 positivity may not be entirely helpful in distinguishing PRCCs from ACD-associated RCCs and HLRCC-associated RCCs, both of which are usually composed of cells with eosinophilic cytoplasm, frequently showing a papillary/tubular pattern. Genetically, gain of chromosomes 7 and/or 17 and loss of the Y chromosome are characteristically associated with PRCCs. According to the comprehensive molecular profiling of PRCCs by TCGA [3], type 1 PRCCs are characterized by *MET* alterations, whereas type 2 PRCCs are characterized by *CDKN2A* silencing, *SETD2* mutations, and increased expression of the NRF2-antioxidant response element pathway. Using multiplatform analyses, type 2 PRCCs have been categorized into three clusters (C2a, C2b, and C2c). C2c PRCCs are CpG island methylator phenotype (CIMP)-associated tumors, characterized by poor survival and *FH* mutations. C2a PRCCs are associated with early stages of tumor development. C2b PRCCs are associated with later stages of tumor development and *SETD2* mutations [3].

### 3.3. Chromophobe RCC (ChRCC)

ChRCCs, comprising 5–7% of all adult RCCs, have a favorable prognosis. Birt–Hogg–Dube syndrome with *FLCN* mutations is associated with a higher incidence of ChRCCs. Macroscopically, the cut surfaces of ChRCCs appear light tan, to brown, to mahogany-brown. Microscopically, ChRCCs are characterized by cells with prominent cell membranes, irregular nuclei with perinuclear halos, and pale to eosinophilic cytoplasm. The eosinophilic variant of ChRCCs shows predominantly smaller eosinophilic granular cells with irregular nuclei and perinuclear halos [1]. Immunohistochemically, ChRCCs are typically positive for KIT and CK7, and negative for CAIX and CD10. Genetically, ChRCCs show losses of chromosomes 1, 2, 6, 10, 13, and 17. Eosinophilic variants may have fewer genetic losses [4]. Somatic mutations in mitochondrial DNA are common. *TP53* and *PTEN* are frequently mutated, in 32% and 9% of ChRCCs, respectively [4]. Recurrent DNA rearrangement breakpoints within the *TERT* promoter region in 10% of examined cases of ChRCCs, which are associated with high *TERT* expression and manifestation of kataegis, represent a mechanism for increased *TERT* expression in these tumors, differing from the point mutations of *TERT* observed in various malignancies [4]. A recent study has demonstrated that metastatic ChRCCs were characterized by *TP53* mutations (58%), *PTEN* mutations (24%), and imbalanced chromosome duplication (ICD) (25%), suggesting these genomic changes are involved in metastatic evolution for ChRCCs [24].

## 4. New Subtypes

In the 2016 WHO classification, seven new subtypes were adopted, as shown in Table 1. The features of their molecular pathological epidemiology are briefly summarized in Table 3.

### 4.1. Multilocular Cystic Renal Neoplasm of Low Malignant Potential

According to the 2004 WHO classification [32], multilocular cystic RCC exhibits an excellent prognosis; therefore, its name was changed to multilocular cystic renal neoplasm of low malignant potential. It is defined as a renal tumor composed entirely of cysts, the septa of which contain individual, or groups of, clear cells without expansive growth [1]. Immunohistochemically, the neoplastic cells are positive for CAIX and CK7, as is the case for CCPRCC [33]. As genetic studies have distinctly linked multilocular cystic RCC with CCRCC [33,34], multilocular cystic renal neoplasm of low malignant potential is similar to CCRCC, not only morphologically but also genetically. Similar to CCRCC, chromosome 3p deletions and *VHL* mutations were found in 74% and 25% of these tumors, respectively [34,35].

### 4.2. MiT Family Translocation RCC (MiT Family TRCC)

Xp11 TRCC was established as an RCC subtype in the 2004 WHO classification [32]. In the 2016 WHO classification [1], MiT family TRCC, composed of Xp11 TRCC and t(6;11) RCC, was newly defined as an RCC subtype. Both Xp11 translocation and t(6;11) RCC are characterized by the rearrangement of the MiT transcription factors, *TFE3* and *TFEB* [36]. Xp11 TRCC comprises 20–40% of pediatric RCCs and 1–4% of adult RCCs, with an average age of onset of 50 years [37,38]. The t(6;11) RCC subtype is very rare, with approximately 60 cases reported to date. The mean and median patient ages are both approximately 30 years [39]. Morphologically, Xp11 TRCC is typically composed of cells with clear/eosinophilic cytoplasm with papillary and nested structures and psammoma bodies. The tumor cells are large with prominent nucleoli [40,41]. The t(6;11) RCC subtype typically shows a biphasic component and is composed of nests of larger epithelioid cells and smaller cells around the basement membrane [42,43]. However, MiT family TRCCs occasionally show diverse morphological patterns without a characteristic morphology [44,45]. Specifically, Xp11 TRCC and t(6;11) RCC display positive nuclear immunostaining for TFE3 and TFEB, respectively [45,46]. Xp11/*TFEB* TRCC is diagnosed by the utilization of TFE3/TFEB immunohistochemistry or break-apart *TFE3*/*TFEB* fluorescence in in situ hybridization in formalin-fixed paraffin-embedded (FFPE) specimens [43,45,46,47,48]. A recent study suggested that the reverse transcriptase-polymerase chain reaction is a highly sensitive and specific method that can be used with FFPE tissues for the diagnosis of Xp11 TRCC [49]. Another recent study demonstrated that *TFEB*-amplified RCCs with or without *TFEB* translocation were characterized by aggressive clinical behavior, variable morphology, aberrant melanocytic marker expression, and high frequency of immunohistochemical positivity for TFEB [50].

### 4.3. Tubulocystic RCC

Tubulocystic RCC is an uncommon cystic RCC, comprising fewer than 1% of all RCCs. Patients have a mean age of 60 years and are predominantly male [51,52,53,54]. Morphologically, tubulocystic RCC is composed of small- to intermediate-sized tubules and cystically dilated larger tubules. The luminal spaces are lined by a single layer of atypical cells with a cuboidal/hobnail configuration. The tumor cells have enlarged nuclei with prominent nucleoli and abundant eosinophilic cytoplasm [1,28]. Immunohistochemically, the tumor is positive for CD10 and AMACR [28]. Tubulocystic RCC shows gains of chromosomes 7 and 17, and loss of the Y chromosome, suggesting similarity with PRCC. Although collecting duct carcinoma occasionally displays areas that morphologically resemble tubulocystic RCC [55], gene expression profiling studies suggest that tubulocystic RCC and collecting duct carcinoma should be considered two distinct entities at the molecular level [56]. Recent evidence has shown that tubulocystic RCC with poorly differentiated foci is strongly associated with an FH-deficient status and aggressive behavior [57].

### 4.4. Acquired Cystic Disease-Associated RCC (ACD-Associated RCC)

ACD-associated RCC is the most common tumor of the kidney in patients with end-stage renal disease and ACD [1,58]. ACD-associated RCC is generally indolent; however, a subset of patients with ACD-associated RCC may suffer from metastatic disease [28,59]. Microscopically, ACD-associated RCC is characterized by tumor cells with eosinophilic cytoplasm, cribriform/sieve-like patterns, and intratumoral oxalate crystals. ACD-associated RCC has variable structures, including papillary, tubulocystic, macrocystic, solid, and cribriform patterns [28,58,60]. The presence of intratumoral oxalate crystals and intracytoplasmic microlumen formation (vacuoles) imparting a cribriform, microcystic, sieve-like pattern, helps distinguish ACD-associated RCC from other renal neoplasms. The tumor cells are typically large with eosinophilic granular cytoplasm, ill-defined cell membranes, and prominent nucleoli [1,28,58]. Immunohistochemically, ACD-associated RCC is typically positive for AMACR and CD10, and negative for CK7 [1]. Genetically, the high prevalence of gains of chromosomes 3, 16, and Y can distinguish ACD-associated RCC from PRCC, with which ACD-associated RCC shows overlapping morphological and immunophenotypic features.

### 4.5. Clear Cell Papillary RCC (CCPRCC)

CCPRCC is likely the fourth most common RCC subtype, accounting for approximately 3–4% of all renal tumors, close in incidence to that of ChRCC [61,62]. CCPRCC is generally indolent, with very rare reports of recurrence or metastasis [61,62,63,64]. CCPRCC is composed of cells with clear cytoplasm and often shows a papillary structure; however, it demonstrates tubular, papillary, and solid patterns of varying proportions [61,63,64]. One of its characteristic morphologies is the presence of apical- to mid-oriented, linearly arranged uniform nuclei. Although this morphology may overlap with low-grade CCRCC/PRCC, the immunohistochemical characteristics of CCPRCC can help distinguish it from low-grade CCRCC/PRCC. CCPRCC is typically positive for CAIX and CK7, and negative for CD10 [61,63,64]. It generally lacks the genomic alterations observed in CCRCC/PRCC; It lacks the 3p deletion, *VHL* mutation, *VHL* promoter hypermethylation, and gain of chromosome 7 or 17 [63,65]. A study examining seven cases of CCPRCC, by comparative genomic hybridization, detected no chromosomal imbalances [66].

### 4.6. Succinate Dehydrogenase-Deficient RCC (SDH-Deficient RCC)

SDH-deficient RCC occurs predominantly in patients with germline mutations in one of the *SDH* genes: *SDHA*, *SDHB* (most common), *SDHC*, or *SDHD*. Autosomal *SDH* genes encode proteins that assemble at the inner mitochondrial membrane to form mitochondrial complex 2 [67]. SDH-deficient RCC comprises 0.05–0.2% of all renal carcinomas, with a mean patient age of 37 years [68]. Metastasis from SDH-deficient RCC is rare [68,69]. Macroscopically, SDH-deficient RCC is usually well circumscribed with a tan to red cut surface [68]. Microscopically, SDH-deficient RCC is either well circumscribed or shows coarse lobulation with cystic changes in the form of microcysts and macrocysts, containing pale eosinophilic fluid. The most distinctive feature of these tumors is the presence of cytoplasmic vacuoles and inclusion-like spaces [68]. Immunohistochemically, SDH-deficient RCC is typically negative for SDHB, which is considered diagnostic for these tumors [1]. SDH-deficient RCC is also typically negative for KIT and CK7, which are usually present in ChRCC and can be used to distinguish SDH-deficient RCC from ChRCC. Genetically, the defining molecular abnormality in SDH-deficient RCC is the double-hit inactivation of one of the *SDH* genes, most commonly *SDHB* [1,68]. A study examining six cases of SDH-deficient RCC by next-generation DNA sequencing showed that none of the examined cases harbored genetic mutations that contribute to RCC pathogenesis, including *VHL*, *PIK3CA*, *AKT*, *MTOR*, *MET*, and *TP53* [70]. A recent study demonstrated that metastatic lethal SDHB-deficient RCCs characteristically exhibit diverse morphologies, including high-grade features, and show an extreme Warburg effect [71].

### 4.7. Hereditary Leiomyomatosis and RCC-Associated RCC (HLRCC-Associated RCC)

HLRCC-associated RCC occurs in patients with HLRCC syndrome, which is an autosomal dominant hereditary disease that is associated with germline mutations in *FH* at chromosome 1q42.3-q43. The characteristic features of this syndrome are multiple cutaneous and uterine leiomyomas and HLRCC-associated RCCs. A diagnosis of HLRCC-associated RCC is confirmed by the presence of germline *FH* mutations. In contrast to other syndrome-associated RCCs, HLRCC-associated RCC often occurs as a solitary mass with aggressive behavior. Thus, the prognosis of HLRCC-associated RCC is poor, with a tendency for early widespread dissemination [1,72]. Microscopically, HLRCC-associated RCC resembles type 2 PRCC and collecting duct carcinoma, is composed of tumor cells with an abundant eosinophilic cytoplasm, and shows a papillary/tubular pattern. The most distinctive feature of HLRCC-associated RCC is the presence of characteristically large nuclei with inclusion-like eosinophilic nucleoli and perinuclear clearing [63,73] Immunohistochemically, HLRCC-associated RCC is typically negative for high-molecular weight cytokeratins (CK19 and 34betaE12) and CK7, which are positive in collecting duct carcinoma, and can be included in a differential diagnosis with HLRCC-associated RCC. HLRCC-associated RCC is typically negative for CK7, which differs from PRCC, and CD10, except in areas with clear cell features [63,73]. Recently, immunohistochemistry to detect FH and S-(2-succino)-cysteine (2SC), whose accumulation is caused by a loss of FH enzymatic activity, has been developed [74]. HLRCC-associated RCC is characterized by negative immunohistochemical results for FH and positive results for 2SC [75]. Genetically, HLRCC-associated RCC is characterized by a germline mutation in *FH*. In HLRCC-associated RCCs, oxidative phosphorylation is impaired; therefore, tumor cells demonstrate a metabolic shift to aerobic glycolysis. In conditions of *FH* deficiency, fumarate increases and becomes an oncometabolite. Increased fumarate impairs HIF prolyl hydroxylase, which results in increased HIF1A levels [1].

## 5. Emerging/Provisional Entities

The 2016 WHO classification identified emerging/provisional new entities that are rare and yet fully characterized by morphology, immunohistochemistry, and molecular analyses, and require refinement of their diagnostic criteria and the revealing of clinicopathological features [1]. The characteristic features of emerging/provisional entities are summarized in Table 4.

### 5.1. Oncocytic RCC Occurring after Neuroblastoma

There is an increased risk of RCC in a patient with neuroblastoma. Oncocytic RCC occurring after neuroblastoma can develop in patients who have a history of neuroblastoma, with or without exposure to chemotherapy [1,76,77,78]. However, renal neoplasms occurring in patients with prior neuroblastoma are thought to represent a heterogeneous group of RCCs, not a single entity [76]. Oncocytic RCC occurring after neuroblastoma demonstrates diverse morphologic features, including an oncocytic appearance that is similar to the typical morphology of MiT family TRCCs. Immunohistochemically, this tumor is positive for PAX8 [76]. Genetically, a subgroup of this tumor demonstrates a *TFE3*/*TFEB* rearrangement [76]. Further studies are required to clarify whether this tumor entity is distinct, with unique histopathological and molecular characteristics.

### 5.2. Thyroid-Like Follicular RCC

Thyroid-like follicular RCC, an extremely rare RCC, resembles thyroid parenchyma with an abundant colloid-like material and is clinically indolent, with rare examples of metastases. The age range is broad, and patients are predominantly female [1,28,79,80,81]. Macroscopically, the tumor typically displays a tan-brown appearance. Microscopically, the tumor resembles thyroid parenchyma with follicles and colloid. Immunohistochemically, the tumor is typically positive for PAX8 and negative for PAX2, TTF-1, and thyroglobulin; TTF-1 or thyroglobulin is required to rule out metastases from thyroid cancer. Little is known about the molecular characteristics of thyroid-like follicular RCC.

### 5.3. ALK Rearrangement-Associated RCC

*ALK* rearrangement-associated RCC occurs in children and adults, with or without sickle cell trait, comprising approximately 0.4% of all adult RCCs [82,83,84,85,86,87,88,89]. In young patients with sickle cell trait, the tumor demonstrates morphological features similar to those of renal medullary carcinoma [86,87]. In patients without sickle cell trait, the tumor morphologically demonstrates papillary, solid, and tubular patterns with prominent nucleoli and eosinophilic cytoplasm, including rhabdoid or signet-ring cell features with psammoma bodies [82,83]. The tumor displays cytoplasmic ALK and nuclear TFE3 immunostaining [82,83,88]. Because *ALK* rearrangement-associated RCC can be immunohistochemically positive for TFE3, such a result should be cautiously interpreted in a differential diagnosis with Xp11 TRCC. Genetically, reported partners of *ALK* are *VCL*, *TPM3*, *EML4*, *STRN*, and *HOOK1* [82,83,86,87,89]. Because *ALK* rearrangement-associated RCCs are potentially responsive to ALK inhibitors [90,91,92,93,94,95], reasonable efforts should be made to identify *ALK* rearrangement-associated RCCs.

### 5.4. RCC with (Angio)Leiomyomatous Stroma

RCC with (angio)leiomyomatous stroma has been recently identified as an RCC subtype, but was historically categorized as either a CCRCC or CCPRCC [1,96,97,98]. This tumor occurs sporadically and can be associated with tuberous sclerosis [99]. Microscopically, the tumor is typically composed of neoplastic glandular structures lined by cells with mixed clear, pale, and eosinophilic cytoplasm, forming occasional papillary structures. The stroma of this tumor resembles smooth muscle and often extends away from the epithelial component [98]. Immunohistochemically, the epithelial component is typically positive for CAIX, CD10, CK7, 34betaE12, and PAX8, and negative for AMACR and HMB45. The stromal component is typically positive for SMA and caldesmon [98]. Genetically, the tumor does not have a 3p deletion or trisomy 7 or 17 [98]. Recent evidence suggests an association of this tumor with *TCEB1* mutations [100].

## 6. Conclusions and Future Directions

This review introduces and briefly summarizes the molecular pathological epidemiology of both new and current subtypes of renal cell tumors, as well as emerging/provisional RCCs, in the new 2016 WHO classification. The increased understanding of morphological, immunohistochemical, molecular, and epidemiological features of renal cell tumors enables us to categorize renal neoplasms into subtypes/entities with distinct characteristics. In the near future, the WHO classification will need to be further revised to allow for reliable, clinically meaningful diagnoses of these tumors. For that purpose, a more detailed and comprehensive understanding of their molecular pathological epidemiologic features is required.

## Figures and Tables

**Table 1:** Classification of renal cell tumors according to the 2016 WHO classification [1].

Current Renal Cell Tumor Subtypes	New Renal Cell Tumor Subtypes
Clear cell RCC	Multilocular cystic renal neoplasm of low malignant potential
Papillary RCC	MiT family translocation RCC
Chromophobe RCC	Tubulocystic RCC
Collecting duct carcinoma	Acquired cystic disease-associated RCC
Renal medullary carcinoma	Clear cell papillary RCC
Mucinous tubular and spindle cell carcinoma	Succinate dehydrogenase-deficient RCC
RCC, unclassified	Hereditary leiomyomatosis and RCC-associated RCC
Papillary adenoma	
Oncocytoma	

MiT, microphthalmia transcription factor; RCC, renal cell carcinoma; WHO, World Health Organization.

**Table 2:** Current major subtypes of renal cell tumors in the 2016 WHO classification [1].

Renal Cell Tumor Subtypes	Clinical Features	Morphological/Immunohistochemical Features	Molecular Features
CCRCC	65–70% of adult RCCs	Clear/eosinophilic cells with thin-walled, staghorn-shaped vasculature; positive for CAIX and CD10, negative for CK7 and AMACR	Loss of function of *VHL*, Chr 3p deletion, inappropriate stabilization of HIFs, genetic mutations in PI3K/AKT pathway, mutations of *SETD2*, *BAP1*, and *MTOR*, aggressive CCRCC demonstrating a metabolic shift
PRCC	15–20% of adult RCCs, type 1 shows a better prognosis than type 2	Papillary structure, foamy macrophages; type 1: scanty cytoplasm; type 2: abundant eosinophilic cytoplasm; positive for CD10, CK7, and AMACR, negative for CAIX	Gain of Chr 7 and/or Chr 17, loss of Chr Y; type 1: *MET* alteration; type 2: *CDKN2A* silencing, *SETD2* mutation; three subtypes according to the TCGA, including CIMP-associated aggressive subtype with an *FH* mutation
ChRCC	5–7% of adult RCCs, favorable prognosis, Birt-Hogg-Dube syndrome with an *FLCN* mutation	Prominent cell membrane, irregular nuclei, perinuclear halo, pale to eosinophilic cytoplasm; positive for KIT and CK7, negative for CAIX and CD10	Loss of Chrs 1, 2, 6, 10, 13, and 17, somatic mutation in mitochondrial DNA, mutations of *TP53* and *PTEN*, imbalanced chromosome duplication (ICD), high *TERT* expression by DNA rearrangement within the *TERT* promoter region with kataegis

CCRCC, clear cell RCC; Chr, chromosome; ChRCC, chromophobe RCC; CIMP, CpG island methylator phenotype; HIF, hypoxia-inducible factor; PRCC, papillary RCC; RCC, renal cell carcinoma; TCGA, The Cancer Genome Atlas; VHL, von Hippel Lindau; WHO, World Health Organization.

**Table 3:** New subtypes of renal cell tumors in the 2016 WHO classification [1].

New Renal Cell Tumor Subtypes	Clinical Features	Morphological/Immunohistochemical Features	Molecular Features
Multilocular cystic renal neoplasm of low malignant potential	Excellent prognosis	Numerous cysts lined by clear cells; positive for CAIX and CK7	*VHL* mutation, Chr 3p deletion
MiT family TRCC	Pediatric to young adult patients, mean age of 30 years	Papillary pattern, psammoma bodies, large epithelioid cells and small cells; positive for TFE3 or TFEB	Xp11 TRCC: *TFE3* rearrangement, t(6;12) RCC: *TFEB* rearrangement
Tubulocystic RCC	Male predominance, mean age of 60 years, indolent	Dilated tubules with a single layer of cells	Gain of Chrs 7 and 17, loss of Chr Y
ACD-associated RCC	End-stage renal disease or ACD, indolent	Eosinophilic cytoplasm, sieve-like pattern, intratumoral oxalate crystals; positive for AMACR and CD10, negative for CK7	Gain of Chrs 3, 16, and Y
CCPRCC	3–4% of renal tumors, indolent, end-stage renal disease, VHL disease	Clear cytoplasm, papillary pattern, apical-oriented nuclei; positive for CK7 and CAIX, negative for CD10	Lack of the genomic alterations observed in CCRCC/PRCC
SDH-deficient RCC	0.05–0.2% of renal carcinomas, mean age of 37 years, good prognosis, germline mutation in one of the *SDH* genes	Cytoplasmic vacuoles and inclusion-like spaces; negative for SDHB, KIT, and CK7	Double-hit inactivation of one of the *SDH* genes, most commonly *SDHB*, no mutations in *VHL*, *PIK3CA*, *AKT*, *MTOR*, *MET*, or *TP53*
HLRCC-associated RCC	HLRCC syndrome, aggressive	Large nuclei with inclusion-like eosinophilic nucleoli and perinuclear clearing, abundant eosinophilic cytoplasm, papillary/tubular pattern; positive for S-(2-succino)-cysteine (2SC), negative for FH, CK19, 34betaE12, and CK7	Germline mutation in *FH*, metabolic shift to aerobic glycolysis, increased fumarate and HIF1A

ACD, acquired cystic disease; CCPRCC, clear cell papillary RCC; CCRCC, clear cell RCC; Chr, chromosome; HLRCC, Hereditary leiomyomatosis and RCC; MiT, microphthalmia transcription factor; PRCC, papillary RCC; RCC, renal cell carcinoma; SDH, succinate dehydrogenase, TRCC, translocation RCC; VHL, von Hippel Lindau; WHO, World Health Organization.

**Table 4:** Emerging/provisional entities of RCCs in the 2016 WHO classification [1].

Emerging/Provisional RCC Subtypes	Clinical Features	Morphological/Immunohistochemical Features	Molecular Features
Oncocytic RCC occurring after neuroblastoma	Increased risk of RCC after prior neuroblastoma, with or without exposure to chemotherapy	Oncocytic appearance, appearance similar to MiT family TRCC; positive for PAX8	A subgroup demonstrates *TFE3*/*TFEB* rearrangement
Thyroid-like follicular RCC	Extremely rare, broad age range, slight female predominance, indolent	Thyroid parenchyma-like appearance with follicles and colloid; positive for PAX8 and negative for PAX2, TTF-1, and thyroglobulin	NA
*ALK* rearrangement-associated RCC	0.4% of adult RCCs, sickle cell trait	With sickle cell trait: renal medullary carcinoma-like morphology; without sickle cell trait: papillary, solid, and tubular patterns with prominent nucleoli and eosinophilic cytoplasm with psammoma bodies; positive for ALK and TFE3	*ALK* rearrangement; reported fusion partners are *VCL*, *TPM3*, *EML4*, *STRN*, and *HOOK1*
RCC with (angio)leiomyomatous stroma	Occurring sporadically or associated with tuberous sclerosis, historically categorized as CCRCC or CCPRCC	Glandular or papillary structures lined by cells with mixed clear, pale, and eosinophilic cytoplasm, stroma resembling smooth muscle; epithelial component: positive for CAIX, CD10, CK7, and 34betaE12, negative for AMACR; stromal component: positive for SMA	Without a 3p deletion, without trisomy 7 or 17, associated with *TCEB1* mutation

CCPRCC, clear cell papillary RCC; CCRCC, clear cell RCC; MiT, microphthalmia transcription factor; NA, not available; RCC, renal cell carcinoma; TRCC, translocation RCC; WHO, World Health Organization.

## Abbreviations

2SC	S-(2-succino)-cysteine
ACD	acquired cystic disease
CCPRCC	clear cell papillary RCC
CCRCC	clear cell RCC
Chr	chromosome
ChRCC	chromophobe RCC
FFPE	formalin-fixed paraffin-embedded
FH	fumarate hydratase
HIF	hypoxia-inducible factor
HLRCC	hereditary leiomyomatosis and RCC
ICD	imbalanced chromosome duplication
ISUP	International Society of Urological Pathology
PRCC	papillary RCC
RCC	renal cell carcinoma
SDH	succinate dehydrogenase
TCGA	The Cancer Genome Atlas
TRCC	translocation RCC
VHL	von Hippel Lindau
WHO	World Health Organization
